# A comparison of mosquito sampling methods in six Pacific Island countries

**DOI:** 10.1016/j.onehlt.2025.101118

**Published:** 2025-06-23

**Authors:** Adam T. Craig, Amanda K. Murphy, Charlie Ave, Nelson Ngaiorae, Lesieli Maha, Filisi Tonga, Charles Butafa, Vineshwaran Rama, Fata Paulo, Tabomoa Tinte, Tessa B. Knox, Holly Jian, Geoff Fisher, Tanya L. Russell, Thomas R. Burkot

**Affiliations:** a Center for Clinical Research, The University of Queensland, Herston, Queensland, Australia; b Australian Institute of Tropical Health and Medicine, James Cook University, Cairns, Queensland, Australia; c Te Marae Ora Cook Islands Ministry of Health, Rarotonga, Cook Islands; d Tonga Ministry of Health, Nuku'alofa, Tonga; e Solomon Islands Ministry of Health and Medical Services, Honiara, Solomon Islands; f Fiji Centre for Disease Control, Ministry of Health and Medical Services, Suva, Fiji; g Samoa Ministry of Health, Apia, Samoa; h Kiribati Ministry of Health and Medical Services, Tarawa, Kiribati; i Beyond Essential Systems, Melbourne, Australia

**Keywords:** Entomology, Dengue, Pacific Islands, Latin square, Arboviral disease, Mosquito sampling, Surveillance

## Abstract

Outbreaks of arboviral diseases pose a significant threat to health security in Pacific Island countries and territories. In the absence of vaccines or treatments, effective vector control is critical to reduce risk and respond to outbreaks. This relies on sustainable mosquito surveillance strategies to identify vectors and guide control efforts. This study evaluated the performance and feasibility of three adult mosquito sampling methods—BG-Sentinel II (BGS) traps, BG Gravid *Aedes* Traps (GAT), and sweep netting (SWN)—in six Pacific countries: Cook Islands, Fiji, Kiribati, Samoa, Solomon Islands, and Tonga. Sampling followed a Latin square design across 54 sites in 18 locations. Data were analysed using a generalised linear mixed model and Simpson's Index for diversity. Qualitative interviews with public health staff captured operational experiences. 2815 mosquitoes were collected, with *Aedes* species comprising 61 %. Species composition varied significantly between countries (*p* < 0.05). BGS traps yielded considerably more mosquitoes than GAT and SWN (p < 0.05). No major species bias was observed across sampling methods. The public health staff interviewed emphasised the value of mentoring, co-design, and resourcing for operational research. Pacific context-specific challenges underscored the need for simple, durable tools for routine use, particularly if to be used in remote settings. This is the first multi-country study conducted in the Pacific to compare *Aedes* sampling methods.

## 1 Background

Arboviral diseases, particularly dengue, Zika and chikungunya, threaten the health security of populations in Pacific Island countries and territories (PICs) [[1], [2], [3]]. Dengue is persistent and possibly endemic in several PICs, including Fiji, French Polynesia, New Caledonia, the Cook Islands, Papua New Guinea, Solomon Islands and Vanuatu [[4], [5], [6]].

The Pacific region is unique in having 13 *Aedes* species capable of transmitting dengue [7]. *Culex* vectors of other viruses such as Ross River virus and Japanese encephalitis virus are also present, though they currently pose a lower public health risk compared to *Aedes-*transmitted dengue, Zika and chikungunya viruses [1,7]. Each vector species exhibits distinct bionomic characteristics, including susceptibility to insecticides, oviposition preferences, as well as biting and resting behaviours [7]. The effectiveness of public health vector interventions depends on the distinct vector behavioural vulnerabilities of each species. For example, *Ae. aegypti*, the primary dengue vector, prefers to bite and rest indoors and thus can be effectively controlled through indoor residual spraying, whereas *Ae. albopictus* and *Ae. polynesiensis* (considered secondary dengue vectors) predominantly bite and rest outdoors and thus will be more vulnerable to outdoor residual insecticide applications. National public health programs aiming to reduce arboviral disease risk must know and target the vectors present where people are likely to be exposed [8]. *Aedes* mosquitoes are known as highly invasive mosquitoes due to their having desiccant-resistant eggs, and consequently, the distributions of the *Aedes* mosquito vectors are continuously changing. Therefore, the first step in implementing effective vector control strategies is understanding the current distributions of the vectors.

Despite the rapid spread of dengue and other arboviral diseases across PICs [7], vector surveillance is limited and sporadic. Insufficient human resources, challenging topographies, limited transport options and lack of equipment constrain countries' capacities for vector surveillance and control and have inhibited effective public health action [9]. These limitations emphasise the need for careful evidence-based planning to ensure that resources available for vector surveillance and control are used to maximum impact. Central to this is choosing the best-performing yet operationally feasible vector surveillance method. Multiple tools for monitoring adult mosquito population presence and abundance have been developed [8], but their performance and operational feasibility in PIC contexts have not been compared systematically.

The Pacific Mosquito Surveillance Strengthening for Impact (PacMOSSI) consortium (www.pacmossi.org) is a multi-partner initiative funded by the Australian, French and New Zealand Governments and the European Union. PacMOSSI supports PICs to strengthen national vector surveillance and control to prevent, contain and manage mosquito-borne diseases, thereby improving the health and wellbeing of Pacific communities [10]. PacMOSSI supports PICs in building the capacity to undertake mosquito surveillance and vector control and developing strategic plans tailored to local contexts. A key PacMOSSI activity is designing and implementing country-led operational research (OR) to collect local data relevant to country strategic planning.

During 2023 and 2024, a multi-country OR study was developed and implemented to compare the performance and operational feasibility of three adult mosquito sampling methods. The OR had two aims: (i) to analyse PIC-generated data on mosquito sampling methods for monitoring mosquito presence and abundance and (ii) to assess the operational practicality of each sampling method for routine public health use. The intent of this project was to build experience and knowledge of public health staff within PIC Ministries of Health (MoH) to perform OR, and, in so doing, build long-term evidence generation capacity to support informed policy decision-making.

## 2 Methods

### 2.1 Setting

**Supplementary Fig. S1:**
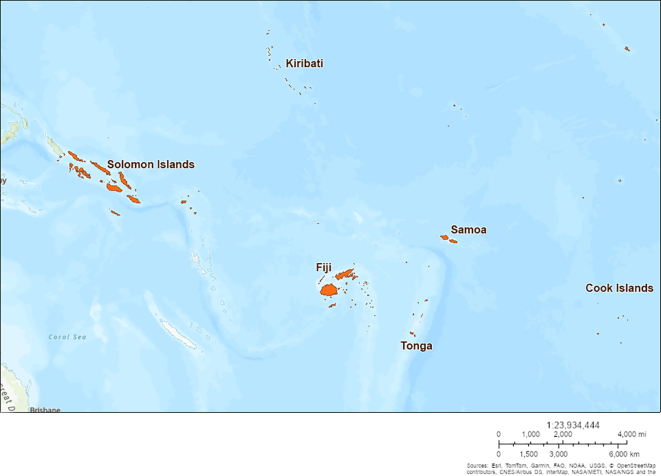
Map of the Pacific Island region highlighting the location of the six participating countries.

The Pacific Islands are distributed across a third of the Earth's surface and are home to 11.4 million people, 8.2 million of whom reside in Papua New Guinea. The remaining population is dispersed across the thousands of islands constituting the other 21 PICs [11]. Eight PICs have populations of less than 25,000, and three have less than 10,000 [11]. All PICs are classified by the United Nations as low-middle income countries, with three (Fiji, Samoa and Tonga) within the “high” human development stratum and four (Kiribati, Papua New Guinea, Solomon Islands and Vanuatu) in the “medium” stratum [12]. The locations of the six PICs in this study are shown in Supplementary Fig. S1.

### 2.2 Training in operational research

PacMOSSI provides training on aedine and anopheline vector surveillance and control through eight self-paced online training modules at no cost to registered users (https://pacmossi.org/online-course/). The modules cover mosquito biology, vector surveillance, vector control, World Health Organization (WHO) guidance for vector control, insecticide resistance monitoring, data management, community engagement and OR. Each module takes 3–5 h to complete. Students undertake knowledge check exercises and quizzes throughout the training.

The OR training module uses a constructivist approach in which students are walked through the steps of developing their own OR projects. The training is supported by an activity-based workbook that, once complete, can be used to draft a study protocol, ethics submission or grant proposal.

### 2.3 Operational research study design

#### 2.3.1 Co-design

At the 2022 PacMOSSI regional meeting, PIC MoH staff acknowledged that data about the distribution of arboviral disease vectors was outdated in most PICs and that technical assistance was required to support entomological surveys. The mosquito surveillance tools currently used across the PICs varied, being selected by availability and familiarity rather than by evidence of efficacy in sampling the mosquito vector species presumed to be present. An outcome of this meeting was a commitment to design and undertake OR to generate Pacific-specific evidence about trapping method performance to inform future national mosquito surveys.

Two months later, each PIC was invited to join a working group to develop a multi-country OR project on mosquito sampling methods, with six countries joining the working group. Over three months in mid-2023, the working group collectively designed OR research questions and developed the study protocol. Virtual working group meetings were held, and participants communicated via email between meetings.

#### 2.3.2 Research questions

The OR sought answers to the following questions: (i) What is the relative sensitivity and specificity of three commonly used mosquito sampling methods in PICs? (ii) How operationally feasible is each sampling method? (iii) How effective was the OR as a training aid for MoH staff?

#### 2.3.3 Sampling methods

Three mosquito sampling methods were compared: BG Sentinel II (BGS) traps, Gravid *Aedes* Traps (GAT) and Sweep Net (SWN) collections.

BG Sentinel II traps consist of a collapsible, dark blue fabric container with a white lid perforated with holes. An electric fan draws air into the trap through a black catch pipe, and captured mosquitoes are retained in a black netting cage. These traps were operated using mains or battery power at each collection location for 24 h, with only the trap as a visual attractant for mosquitoes.

Gravid *Aedes* Traps are black plastic containers partly filled with water incorporating an organic attractant (to mimic natural oviposition sites). The attractant was made of rainwater to which grass was added and allowed to ferment for 24-h before being added to the trap. The GAT has a lid with fine mesh, allowing mosquito entry but preventing escape. Oil or insecticide was applied on the inside surfaces of the GAT to incapacitate mosquitoes. The GAT operated for 24-h at each collection location.

Sweep Net collections involved the use of 40 cm diameter circular sweep nets swung in a unidirectional figure-of-eight pattern near the mosquito collector for 5 min within an hour after dawn or before dusk. The mosquito collectors were instructed to wear long sleeves and trousers to minimise exposure to mosquito bites.

#### 2.3.4 Experimental design

A 3 × 3 Latin square evaluation of the three mosquito sampling methods was implemented at three sites in the six participating PICs (Supplementary Fig. S1). Each site was an urban or peri-urban village, at least 1 km from another site. At each site, three outdoor sampling locations were selected, each at least 50 m from any other sampling location and within 10 m of an inhabited dwelling.

The three sampling methods were cycled through each sampling location, with one Latin square considered complete after each method had been implemented at each sampling location for all sites in a PIC. This was repeated until three Latin squares were completed at all sites. Sampling was conducted between October 2023 and January 2024 when weather conditions are hot and wet across the participating PICs.

A Latin square experimental design is frequently used in entomological research to compare mosquito sampling strategies under conditions where multiple environmental and temporal factors may affectperformance. Owing to its methodological simplicity, this design was considered contextually appropriate.

#### 2.3.5 Mosquito identification and diversity

At the end of each sampling event, collected mosquitoes were transported to a laboratory in a container labelled with the date, sampling location and a unique trap identification code. Samples were frozen before morphological identification was performed using a light microscope and a pictorial identification key of the vectors of the Pacific [13]. Mosquito data were recorded in a purpose-built database. The data recorded included sampling location, date, time and method, mosquito genus, species and sex and environmental conditions at the time of collection.

Using the R package “vegan” [14], Simpson's Diversity Index was calculated to compare mosquito species capture diversity. The index is calculated as:

$$D=1-\sum_{i=1}^{s}\left(\frac{n_{i}\left(n_{i}-1\right)}{N\left(N-1\right)}\right)$$

Where $n_{i}$ = the number of species; N = the total number of mosquitoes in the sample and S = the total number of categories observed.

The index scale was inverted so that high diversity is indicated by a result close to 1 and low diversity close to 0.

#### 2.3.6 Interview participant recruitment and interviews

Once all collections were completed for a country, two researchers (AC and AM) conducted semi-structured interviews with PIC staff who led the project in their respective jurisdictions. Invitations were sent to staff in PICs. Interviews were conducted virtually over Zoom and took 30 to 60 min. Non-respondents to the invitation were followed up 2-weeks and then, if needed, 3-weeks after the initial contact. An interview guide based on the 2022 updated version of the Consolidated Framework for Implementation Research (CFIR) [15] facilitated discussions. As per the CFIR, the guide framed interview discussions around external (i.e., outside of the interviewee's immediate sphere of influence) and internal (i.e., inside the interviewee's sphere of influence) factors influencing the ability to implement vector surveillance and OR, as well as personal and project characteristics. Detailed notes were taken with participants' responses captured verbatim where possible.

### 2.4 Mosquito distribution

As *Aedes* species distributions and species collections differed among the six countries, analysis was stratified by the presence of *Aedes* species. The primary *Culex* species present did not differ among the six countries. Table 1 shows each PIC's primary *Aedes* and *Culex* vector species. For a full list of vector species known to be present in each PIC, see *A Guide to Mosquitoes of the Pacific* [7].

**Table 1:** Arboviral disease vector species in the study countries.

Pacific island countries	Key vector species present				
	*Ae. aegypti*	*Ae. albopictus*	*Ae. polynesiensis*	*Cx. quinquefasciatus*	*Cx. annulirostris*
Cook Islands	●		●	●	●
Fiji	●	●	●	●	●
Kiribati	●	●		●	●
Samoa	●	●	●	●	●
Solomon Islands	●	●		●	●
Tonga	●	●		●	●

Source [7].

### 2.5 Analysis

#### 2.5.1 Statistical analysis

All experiment data were collected on hardcopy before entry into a purpose-built electronic database (www.bse.com.au) using a smartphone-based electronic data entry form.

Data were exported and cleaned in Microsoft Excel before being analysed in R [16]. Descriptive statistics were performed using the sampling method and location to estimate percentages, arithmetic means and 95 % confidence intervals (CIs) for each mosquito genus and species collected. Functions from R package “dplyr” [17] were used for analysis.

A negative binomial generalised linear mixed model (GLMM) compared mosquito counts by the different sampling methods [18]. The model included the sampling method as a fixed effect, with country, collection location, Latin square round and sampling time as random effects. The R package “glmmTMB” was used to develop the GLMM [19].

The SWN data were grouped into collections between 6 am and 7 am or 5 pm and 6 pm –near civil dawn or dusk (i.e., circa 5:54 am - 6:15 am and 6:18 pm - 6:40 pm in Kiribati, the northernmost PIC in the study; and 5:23 am - 5:48 am and 7:08 pm - 7:33 pm in Cook Islands, the southernmost PIC involved in the study) and those performed at other times. We used these collection time groups as variants in our analysis to assess the effect SWN sampling time had on the method's performance. The R package “ggplot2” [20] was used to generate a figure comparing the performance of SWN by collection time.

#### 2.5.2 Qualitative analysis

Interview-based qualitative data were analysed using a deductive thematic approach following the process described by Terry et al. (2017). This involved the two interviewers (AC and AM) discussing the interviews and comparing their notes to become familiar with the data, iterative coding of the data, theme identification, revision and refinement, descriptive naming of themes, and reporting [21,22].

## 3 Results

The study was implemented at 54 sampling locations across 18 sites in 6 countries. Of the targeted 162 sampling events per sampling method, we collected data from 186 (114.8 %) SWN events, 155 (95.6 %) BGS, and 152 (93.8 %) GAT collection events. Over-sampling of SWN was due to deviation from the protocol by collectors at some sites (i.e., performing collections in the morning and evening when only one was required). The average of the two collections performed on a single day was calculated in such instances. The under-sampling of SWN and BGS collection events was due to trap failure due to disturbance, or, in one instance, traps being washed away by floodwater.

### 3.1 Mosquitoes sampled

Of the mosquitoes sampled, 1715 (60.7 %) were three *Aedes* species, and 1100 (39.3 %) comprised two *Culex* mosquitoes. In Fiji and Samoa, where *Ae. aegypti*, *Ae. albopictus*, and *Ae. polynesiensis* are present; 924 mosquitoes were sampled with 22.0 % being *Ae. aegypti*, 29.2 % being *Ae. albopictus*, 29.8 % being *Ae. polynesiensis*; 19.0 % were *Culex* spp. In Kiribati, Solomon Islands and Tonga, where *Ae. aegypti* and *Ae. albopictus* are endemic, 35.5 % of the 1745 mosquitoes sampled were *Ae. aegypti* and 14 % were *Ae. albopictus;* the remaining half were *Culex* spp. The Cook Islands harbours both *Ae. aegypti* and *Ae. polynesiensis* and of the 146 mosquitoes sampled there, 26.3 % were *Ae. aegypti*, 24.7 % were *Ae. polynesiensis* and 39 % were *Culex* species (Table 2).

**Table 2:** Count of mosquitoes sampled across all sites by mosquito species and sampling method.

Trap type	Mosquito species	PICs^a^ with *Ae. aegypti, Ae. albopictus* and *Ae. polynesiensis* present		PICs with *Ae. aegypti* and *Ae. albopictus* present			PICs with *Ae. aegypti* and *Ae. polynesiensis* present	Total
		Fiji	Samoa	Kiribati	Solomon Islands	Tonga	Cook Islands	
BG-Sentinel	*Ae. aegypti* (female)	11	91	120	6	13	17	258
	*Ae. aegypti* (male)	3	68	149	5	6	16	247
	*Ae. albopictus* (female)	7	95	39	15	3	..	159
	*Ae. albopictus* (male)	6	68	46	6	0	..	126
	*Ae. polynesiensis* (female)	0	129	..	..	..	23	152
	*Ae. polynesiensis* (male)	0	108	..	..	..	3	111
	*Cx. quinquefasciatus* (female)	8	71	100	17	56	15	267
	*Cx. quinquefasciatus* (male)	0	88	136	30	43	7	304
	*Cx. annulirostris* (female)	2	0	0	0	0	2	4
	*Cx. annulirostris* (male)	2	0	0	0	0	3	5
Gravid *Aedes* Trap (GAT)	*Ae. aegypti* (female)	0	21	30	25	0	9	85
	*Ae. aegypti* (male)	4	2	27	6	0	2	41
	*Ae. albopictus* (female)	35	17	28	32	0	..	112
	*Ae. albopictus* (male)	8	1	14	14	0	..	37
	*Ae. polynesiensis* (female)	15	6	..	..	..	3	24
	*Ae. polynesiensis* (male)	0	12	..	..	..	1	13
	*Cx. quinquefasciatus* (female)	0	2	53	12	2	5	74
	*Cx. quinquefasciatus* (male)	0	0	125	11	2	5	143
	*Cx. annulirostris* (female)	0	0	0	0	0	0	0
	*Cx. annulirostris* (male)	0	0	0	0	0	0	0
Sweep net	*Ae. aegypti* (female)	0	1	91	15	2	5	114
	*Ae. aegypti* (male)	1	5	116	6	2	4	134
	*Ae. albopictus* (female)	17	5	0	34	0	..	56
	*Ae. albopictus* (male)	12	0	6	19	0	..	37
	*Ae. polynesiensis* (female)	2	1	..	..	..	3	6
	*Ae. polynesiensis* (male)	0	0	..	..	..	3	3
	*Cx. quinquefasciatus* (female)	0	0	119	15	12	6	152
	*Cx. quinquefasciatus* (male)	0	0	106	25	6	8	145
	*Cx. annulirostris* (female)	0	0	0	0	0	3	3
	*Cx. annulirostris* (male)	0	0	0	0	0	3	3

a PIC = Pacific Island countries and territories.

Across all countries and sites, 456 (52.3 %) of the *Ae. aegypti* sampled were females. In comparison, 322 (61.7 %) of the *Ae. albopictus* and 181 (58.8 %) of the *Ae. polynesiensis* mosquitoes sampled were females. The BGS trap caught 47.5 % of all female *Aedes* mosquitoes, but the highest female-to-male ratio was observed in GAT traps (2.4:1).

The most abundant *Culex* species sampled was *Cx. quinquefasciatus*, with 1085 individuals captured (98.6 % of all *Culex* mosquitoes). Of these, 45.4 % were females (Table 2). For *Cx. quinquefasciatus*, the BGS trap caught the highest number of females overall (52.6 %), and the highest female-to-male ratio was observed in SWN (51 %).

**Table 3:** Number of sampling events by country, sampling method and (for sweep netting) collection time.

Pacific island countries	BG-Sentinel	Gravid *Aedes* Traps	Sweep net collection			
			Near dawn (6 am–7 am)	Near dusk (5 pm–6 pm)	Other (7 am-5 pm)	All SWN
Cook Is.	27	27	25	0	3	28
Fiji	22	20	0	0	16	16
Kiribati	27	26	24	17	1	42
Samoa	28	27	0	0	27	27
Solomon Is.	27	28	2	13	11	26
Tonga	24	24	12	0	35	47
Total	155	152	63	30	66	186

Fifty-one (32.1 %) of SWN collections were performed between 6 am and 7 am; 22 SWN collections were conducted (13.8 %) between 7 am and 8 am; 27 (17.0 %) between 8 am and 9 am; 1 (0.6 %) between 9 am and 10 am; 16 (10.1 %) between 4 pm and 5 pm; and 42 (26.4 %) between 5 pm and 6 pm. The time of collection was not recorded for 27 SWN collection instances (Table 3).

### 3.2 Comparative performance of sampling methods

**Fig. 1:**
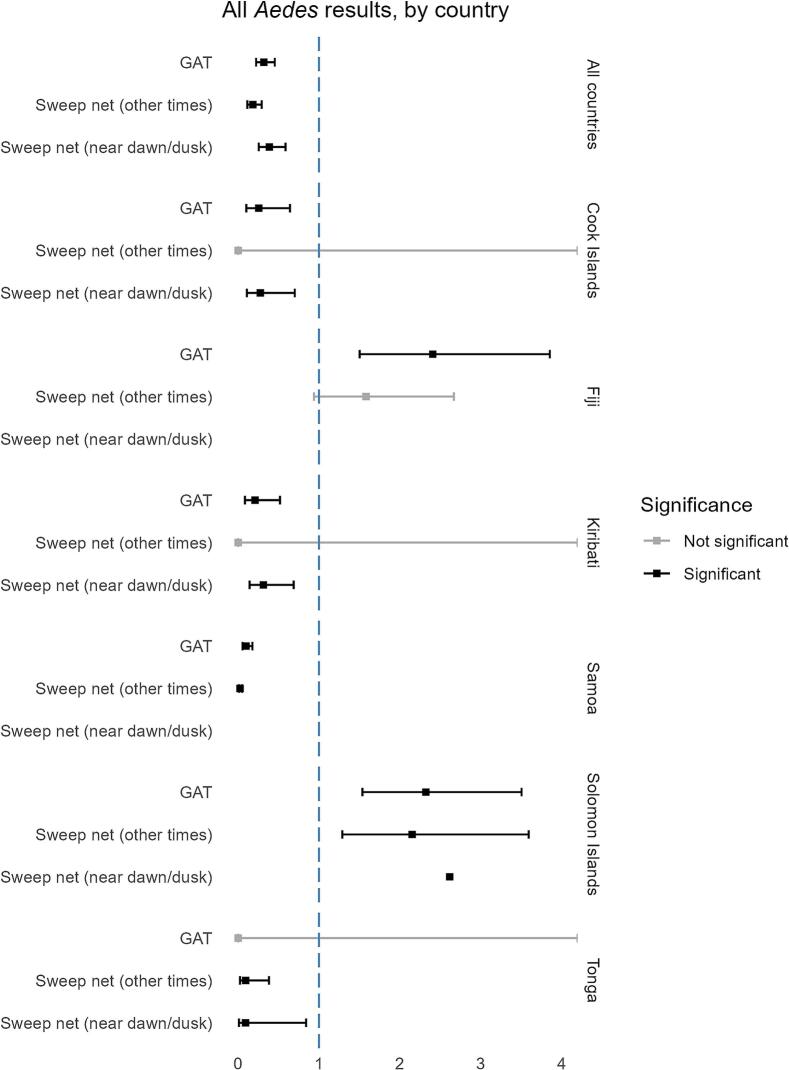
Generalise Linear Mixed Model results for all *Aedes* mosquitoes, all countries and by country. Significance relative to BG Sentinel trap performance (Ref = 1).

**Fig. 2:**
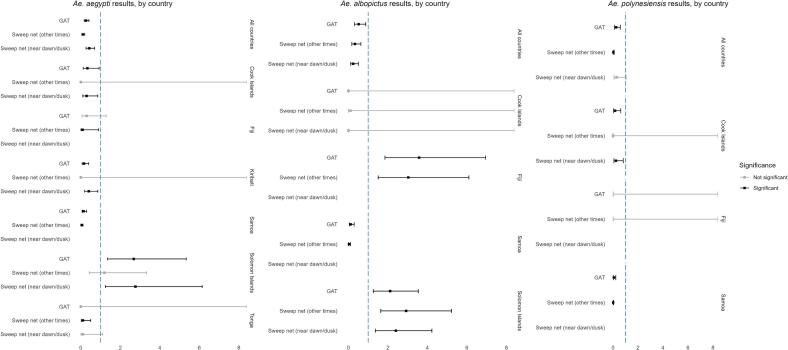
Generalise Linear Mixed Model results for *Ae. aegypti* (left), *Ae. albopictus* (centre) and *Ae. polynesiensis* (right), all countries and by country. Significance relative to BG Sentinel trap performance (Ref = 1).

The exponentiated negative binomial GLMM analysis results by mosquito species and country are shown in Fig. 1, Fig. 2.

**Fig. 3:**
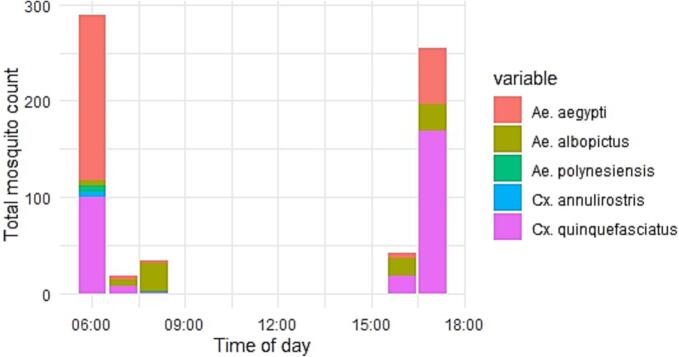
The number and species of mosquitoes sampled using sweep net method, by hour of collection.

When combining data from all sampling locations for all *Aedes* species, BGS traps (Est: [ref = 1]) performed significantly better (*p* < 0.05) than GAT (Est: 0.43 [CI: 0.27–0.70]) and SWN near dawn or dusk (Est: 2.24 [CI: 0.12–0.50]) (Fig. 3). Some variance was observed by country, particularly in Fiji, Tonga and the Solomon Islands, where GAT traps and, in some instances, SWN outperformed the BGS. For example, GAT and SWN methods in Fiji caught three to three-and-a-half-fold more *Ae. albopictus* mosquitoes compared to BGS traps, suggesting significantly better performance. Similar observations were found in the Solomon Islands, where GAT and SWN captured two to three times more *Ae. albopictus* mosquitoes, and in Tonga, GAT traps yielded two and a half times more mosquitoes than the BGS (Fig. 2).

**Supplementary Fig. S2:**
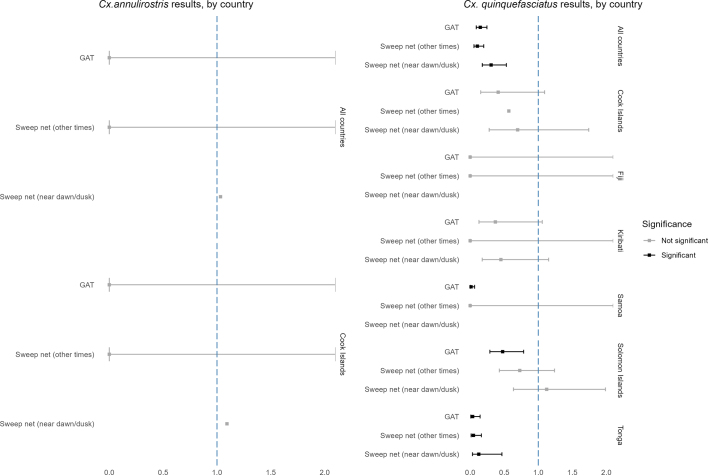
Generalised Linear Mixed Model results for *Cx. quinquefasciatus* (left) and *Cx. annulirostris* (right) mosquito species, all countries and by country.

Counts of *Culex* mosquitoes were typically too small to allow country-level analysis. Still, when data from all collection sites across all countries were pooled, the general observation was that BGS outperformed other methods for collecting *Cx. quinquefasciatus* (*p* < 0.05) and *Cx. annulirostris* (*p* > 0.05) (Supplementary Fig. S2).

### 3.3 Sample diversity

The Simpson's diversity index calculation found that SWN collections sampled a more diverse range of mosquito species than GAT (0.87 (95 % CI: 0.88–0.86) and BGS (0.35 (IQR: 0.30–0.43)).

SWN collections conducted close to dawn or dusk captured a more extensive and diverse range of mosquitoes than those performed at other times (Fig. 3).

### 3.4 Experience implementing the OR project

Nine participants were interviewed to understand their experiences implementing the OR project. Respondents indicated that instigating the OR in the Pacific required individuals experienced in research to steer the study and provide training, as MoHs do not universally have the mandate, skills, or capacity to plan and conduct research independently or organise multi-country activities without outside support. In this study, PacMOSSI consortium partners were from academic institutions with the skills, resources and mandate to conduct research. One respondent noted that there are few Pacific-based researchers and, as such, an understanding of what is involved in setting up and implementing a research project is limited. Another PIC interviewee added that due to the insufficient domestic infrastructure and funding to support research, and that PICs often rely on support from external groups (such as PacMOSSI) to conduct research.

Respondents noted that political support for a new initiative is often limited by funding availability and, as a result, health programs in PICs tend to be vertical (i.e., issue or program-specific) in their design. Most of those interviewed reported that the system strengthening aligned ‘investment’ of the PacMOSSI program over an extended period enhanced leaders' engagement in finding solutions to address the challenges faced in implementing national vector surveillance and control programs. Respondents indicated that the investment was opportunistically timed as awareness of the threat that mosquito-borne diseases pose has been generated by the increased number of arboviral disease outbreaks affecting PICs in recent years.

Beyond the political will leveraged by PacMOSSI funding, respondents reported that the program provided equipment (i.e., sampling tools, microscopes) and funds to secure staff time, ensuring that the resources required to implement the OR study were available. The provision of resources and funding also helped program managers overcome internal human resource and stock procurement challenges that, according to those interviewed, would likely have made independent implementation of the OR project impossible. As one example, a respondent noted that the funding provided supported hiring vehicles and thus allowed staff to get into the field when government vehicles (the usual means of transport) were not available (e.g., early in the mornings or on weekends). Another respondent noted that project funds enabled their ministry to “bring in” junior staff from rural areas to participate in the OR study as a training opportunity. Speaking in general terms, an interviewee said, “Without project funding, we couldn't do activities like this. We'd have to miss out.”

Universally, the interviewees reported value in the iterative co-design approach used to develop the OR project and protocol, noting that the process fostered buy-in and commitment and was, itself, a learning experience. One interviewee captured this sentiment well: “I had no experience with research before this project and didn't know how to go about it. Being involved in the discussion about what we would do and why made me realise what it [research] is all about and why there is such a focus on data collection: getting the forms right and all that.”

Interviewees from five of the six participating PICs indicated that they were generalist environmental health officers with duties broader than vector surveillance, noting that their vector surveillance roles were largely procedural, based on the established practices of the national health authority. For instance, respondents from one PIC noted that vector surveillance is not a routine activity conducted in their country; rather, it is “something we do on an *ad hoc* basis and only in a few sites.” The interviewee said that the national environmental health team do not have any dedicated vector control staff; instead, everyone “knows a little bit.” He added that “staff have experience using GAT traps, and some are familiar with BGS, but no one [has] used SWN before.” No participating PIC had experience with all three sampling methods evaluated in the OR project.

The reason for using (or preferring to use) one sampling method over others was reported to be based on what has, historically, been available and, hence, what was familiar. Responses were mixed when asked how staff used unfamiliar sampling tools as part of the OR study. Generally, the training provided (via the PacMOSSI online modules) was appreciated and felt adequate; however, some challenges were noted. For example, where the study protocol differed from a PIC's routine practice (e.g., the ‘formula’ for preparation of GAT lures, the requirement to place BGS outdoors, or the need to perform sweep netting for exact periods), resulted in errors in trap placement and operation; this may have influenced trap performance.

The opportunity to develop new skills and participate in a multi-country initiative supported by a known development partner (i.e., the PacMOSSI consortium) was highlighted by interviewees as a significant motivator for joining the project and a commitment to see it through to the end of implementation. Universally, respondents conveyed gratitude for the quality and accessible nature of the PacMOSSI training, noting that its content was tailored to reflect the Pacific context and that this enhanced its relevance, enjoyment and learning impact. The link between the OR project and the online OR training objectives was not universally understood. Still, there was broad support for incorporating practical “hands-on” components to contextualise and practice the theoretical content covered in the asynchronous online learning materials. One participant captured this succinctly by saying, “We in the Pacific learn best through doing. We get to see why what is taught is important and get to ask questions … it helps us retain new knowledge and skills.”

Interviewees raised specific challenges with study implementation; some were expected, as previously documented [[23], [24], [25]], but others were not pre-identified. Issues with access to a stable electricity supply to run the BGS fans (whether using mains power or batteries) were expected and experienced, as was the challenge in procuring and transporting heavy batteries and charging equipment. Interviewees reported that BGS were cumbersome, fragile and more challenging to fix in the field. There were safety concerns (trip hazards, electrocution) when electricity cables were required to run across communal and wet outdoor areas to power the trap. SWN collections were often done at times outside of what was stated in the study protocol, as many officers were not able to get into the field before and after regular work hours due to family commitments (e.g., having to get children ready for school) or reliance on government-provided or public transport.

## 4 Discussion

Understanding the composition, ecologies and behaviours of *Aedes* vector species is crucial for preventing dengue, Zika and chikungunya transmission and outbreaks. Effective mosquito surveillance is only possible when operationally feasible and sensitive vector sampling tools are used. Using a Latin square design, this study compared three commonly used mosquito sampling tools (BGS, GAT and SWN) in six PICs and found that, overall, the BGS had a statistically significant higher mosquito trapping efficiency for *Aedes* mosquitoes than the GAT and SWN methods. The performance of GAT and SWN sampling, when performed close to dawn and dusk, was comparable (albeit less effective than BGS).

Differences in trap performance and feasibility were noted between individual countries. While BGS traps outperformed other sampling methods, they were the most logistically challenging to use, requiring either access to a stable electricity supply or the need to purchase, transport and charge direct current batteries, as well as the cumbersomeness and fragility of the trap. There were also safety concerns about operating electrical equipment for BGS traps in a wet environment. These issues raise questions about the utility of the BGS-based sampling method for routine mosquito surveillance in PICs. Conversely, BGS traps may be better suited for short surveys in urban centres when the infrastructure for their use is more accessible. Examples of when this may be the case include research projects, baseline surveys and when identifying vectors present during outbreaks.

The observation that SWN collections performed at dawn or dusk had a similar trapping efficiency to GATs suggests that this low-tech/low-cost sampling method may offer an alternative to the GAT for routine surveillance in the Pacific and in certain situations, such as during outbreaks when insights need to be generated quickly or when in rural and remote areas where it may not be feasible to operate bulky or electrically powered traps. To mitigate the risk of infection to collectors during outbreaks, collectors should wear clothes that cover bare skin (i.e., enclosed shoes, long pants and long-sleeved shirts) and a tropical repellent. The use of SWN also posed logistical challenges associated with getting staff to sampling sites outside normal working hours (dawn and dusk) and community acceptance of sampling activities at these times. The short time window during which mosquitoes in sufficient numbers are likely to be caught by SWN was another challenge as it meant relatively large staff numbers were required to collect samples at multiple villages simultaneously if data collection across dispersed geographical areas is required. Further OR is needed to determine when and how SWN implementation is feasible. Opportunities for citizen participation in mosquito collection using the simple-to-use SWN method is a strategy worth considering to overcome the logistic hurdles associated with government-officer-dependent sampling. Further, citizen participation may address coverage and cost-effectiveness concerns that often impede adequate vector surveillance and—through active involvement in an activity of personal relevance—impact individual and community-level arboviral disease risk knowledge, attitudes and practices.

Five vector species were captured across the collection sites; this does not reflect the full diversity of mosquito species present in each country but rather reflects the types of traps used and the locations in which they were set. An ecological research frame should be adopted to profile species diversity comprehensively, likely involving a more diverse sampling strategy.

The end-of-activity interviews suggested that participating in the design and execution of an OR project was rewarding and enjoyable, and the experience benefited individuals' professional learning. This feedback will inform the PacMOSSI consortium's teaching and learning approach. Specifically, the feedback suggests a constructivist epistemological [26] approach to teaching that creates opportunities for participants to develop knowledge and construct meaning through practical activity and interactions with others is warranted.

The OR project was occasionally modified in response to local needs and circumstances. While these adjustments were well-intentioned, they were made without fully considering their impact on data quality, comparability and analysability. This highlights an important reality that should not be overlooked in future service delivery-focused, officer-led OR initiatives; it also flags a valuable learning opportunity. While ministry personnel bring vital expertise in their field and an understanding of the local context that is invaluable in research, they typically lack formal and comprehensive training in research methods and have had limited opportunities to develop investigation expertise, leading to an underappreciation for the need for (and how to maintain) adherence to study protocols. Bridging this gap through linking theory-based training with opportunities to develop experience through an academic-supported co-design and implementation process proved effective for this project in that it provided time for dialogue about methodological choices; the collective identification and assessment of challenges and the development of strategies to resolve them; and group reflection on the process and learning achieved through the OR project.

This observation highlights the value of authentic collaborations between academic institutions and public health practitioners. Within academic-practitioner partnerships, academia has a key role in guiding and mentoring practitioners to develop the skills to design and implement OR projects that address pressing local policy and program challenges. Such capacity-building efforts can strengthen reflective practice, foster critical thinking and promote rigorous methodological approaches to OR and program design. Conversely, academic-practitioner partnerships also afford valuable opportunities for practitioners to guide researchers by identifying and prioritising areas of high operational relevance. Practitioners serve as vital conduits of the perspectives of healthcare consumers, ensuring that the public's voice informs research. Practitioners also provide a 'reality check' on what will be operationally feasible to implement and how the knowledge gained from OR may best be translated into policy-relevant information. This bidirectional exchange can strengthen the alignment of research with real-world needs and foster a shared commitment to advancing evidence-informed practice.

This study was not without its challenges. First, being implemented across multiple countries, each with its language and customs, meant that nuances in communication might have been missed or meaning misconstrued. Second, the multi-country design meant that inter-rater reliability checking was impractical and not conducted. Third, staff not directly involved in the OR design were engaged in implementing the Latin square experiment; it is unknown how comprehensive the training they received was or if the rigour with which they followed the study protocol was monitored. Fourth, research and research concepts were new to some; hence, insights typically developed through experience may not have been available. Despite these limitations, the study provided valuable contributions. It was the first multi-country comparison of commonly used mosquito sampling methods in PICs, generating regional and vector-specific insights to inform country vector surveillance practices. Further, the study highlights the potential of participatory research as both a methodological approach for knowledge generation and a capacity-building tool, fostering teaching, learning and engagement.

## 5 Conclusion

This study is the first multi-country comparison of commonly used mosquito sampling tools in PICs. It found that BGS outperformed GAT and SWN methods and that the performance of GAT and SWN collection done near dawn and dusk was comparable. The study highlights the value of participatory action research as an approach to addressing practice-related questions and as an effective approach to workforce capacity-building.

The following are the supplementary data related to this article.

## Funding

This work was funded by the Australian Government through a Partnership for a Healthy Region Grant awarded to James Cook University. AC is supported by The University of Queensland‘s Health Research Accelerator (HERA) initiative (2021–2028).

## CRediT authorship contribution statement

**Adam T. Craig:** Conceptualization, Data curation, Formal analysis, Investigation, Methodology, Project administration, Validation, Visualization, Writing – original draft, Writing – review & editing. **Amanda K. Murphy:** Conceptualization, Data curation, Formal analysis, Investigation, Methodology, Writing – original draft. **Charlie Ave:** Data curation, Writing – original draft. **Nelson Ngaiorae:** Data curation, Writing – original draft. **Lesieli Maha:** Data curation, Writing – original draft. **Filisi Tonga:** Data curation, Writing – original draft. **Charles Butafa:** Data curation, Writing – original draft. **Vineshwaran Rama:** Data curation, Writing – original draft. **Fata Paulo:** Data curation, Writing – original draft. **Tabomoa Tinte:** Data curation, Writing – original draft, Writing – review & editing. **Tessa B. Knox:** Writing – original draft. **Holly Jian:** Formal analysis, Visualization. **Geoff Fisher:** Visualization. **Tanya L. Russell:** Formal analysis, Funding acquisition, Methodology, Supervision, Writing – original draft. **Thomas R. Burkot:** Funding acquisition, Methodology, Project administration, Supervision, Writing – original draft.

## Declaration of competing interest

The authors declare that they have no known competing financial interests or personal relationships that could have appeared to influence the work reported in this paper.

## Data Availability

Data will be made available on request.
